# Impacts of COVID-19 pandemic through decomposition of life expectancy according to leading causes and place of death in Czechia

**DOI:** 10.1038/s41598-023-47949-1

**Published:** 2023-11-25

**Authors:** Klára Hulíková Tesárková, Dagmar Dzúrová

**Affiliations:** 1 Department of Demography and Geodemography, Faculty of Sciences, Charles University, Prague, Czechia; 2 Department of Social Geography and Regional Development, Faculty of Sciences, Charles University, Prague, Czechia

**Keywords:** Public health, Cardiovascular diseases, Infectious diseases

## Abstract

While the direct effects of the pandemic are well documented, less is known about the indirect ones, including changes in healthcare provision or human behavior. This paper aims to study the impact of indirect consequences on mortality, focusing on two leading causes (cardiovascular diseases, COVID-19) and places of death in Czechia, during the COVID-19 pandemic, one of the most severely affected European countries. The analysis was performed using data from the Czech Statistical Office and the Institute of Health Information and Statistics. The study compares annual mortality changes during three time periods: pre-pandemic (2018–2019), pandemic beginning and peaking (2020–2021), and pandemic fading (2022). Pandemic years were covered by the WHO public health emergency of international concern. Abridged life tables were computed, and Pollard's decomposition was used to calculate the contributions of causes and places of death on annual differences in life expectancy. Seasonal decomposition of monthly time series revealed an increase in cardiovascular mortality at home or in social care facilities corresponding to limitations in healthcare. While COVID-19 had a systemic negative effect on life expectancy during the pandemic, the impact of cardiovascular mortality according to place of death changed over time. This study contributes to the evidence base of systemic risks during health crises and emergency response.

## Introduction

The COVID-19 pandemic, which began in early 2020, disrupted the global trend of annual increases in life expectancy. Registered COVID-19 deaths were the major contributor to the decline in life expectancy during 2020–2022. While many Western European countries managed to recover from the loss of life expectancy back to pre-COVID life expectancy in 2021, the mortality crisis worsened in many Eastern European countries from 2020 to 2021^1^.

Except for the direct effect of the pandemic (increase in mortality because of COVID-19), the indirect effect of the COVID-19 pandemic (mortality increase because of other reasons and causes than COVID-19 only) occurred in many countries in the world, not only the developed ones. Among others, it led to a decrease in health service utilization^2,3^. It could be supposed that this decrease was driven by a reduced supply of health care (e.g., reorganization of health care and/or medical staff) as well as decreased demand even for acute health care due to, for example, lockdowns, lack of information, or fear of patients in general^4^. In some countries, an increase in mortality from other causes than COVID-19 was observed^5^. As could be expected, this mortality increase is observable above all for cardiovascular diseases (hereinafter “CVDs”), which occur more acutely or need acute medical care^2,3^. Structural changes according to places of death were confirmed as well^5–7^, although this type of analysis is not commonly performed. An increase in CVDs mortality at home or in care homes could be understood as a logical conclusion of limited healthcare usage in hospitals because of many reasons mentioned above.

This study aims to extend the findings with a deeper analysis of the indirect consequences of the COVID-19 pandemic on CVDs mortality and its inner structure according to places of death as well as the timing of the changes during the pandemic. In accordance with the other studies, the development will be described on an annual basis. However, the analysis will focus also on a more detailed point of view, as the specifics related to changes in the pandemic burden and policy responses to it could be better illustrated and discussed in relation to the mortality development. The detailed demographic-epidemiological analysis of Czechia, a country severely affected by the pandemic, helps to enrich the knowledge of how the mortality structure according to the main causes of death was affected by the outbreak of the pandemic when COVID-19 became one of the most important causes of death. To our knowledge, there is insufficient evidence base about mortality structure according to the place of death and its changes during the COVID-19 pandemic, above all in relation to the relevant healthcare provision and usage.

On May 5th, 2023, the WHO Director-General declared that COVID-19 was no longer considered a public health emergency of international concern (“PHEIC”), which was active from January 30th, 2020^8^. Nonetheless, it is crucial to provide an epidemiological investigation of the past and actual health situation for better and more effective preparation for the future.

The pandemic development in Czechia expresses similar features as in the other European countries. Czechia started to monitor COVID-19 cases during the spring of 2020. The most severe peak of the pandemic lasted from autumn 2020 to the end of spring 2021^9^. The last significant increase in COVID-19 mortality came in the second half of 2021 and lasted until spring 2022. On the other hand, there are some important specifics related to the pandemics in Czechia. Above all, Czechia is among the countries where life expectancy decreased not only in 2020 but also in the following year. In many Western European countries, the year 2011 already brought a return to (almost) pre-pandemic mortality levels. In Europe, except for Czechia, the continuation of the mortality worsening was observable in Eastern Europe or in Hungary and Poland^1^. Moreover, Czechia belonged among the most severely affected countries worldwide according to the number of deaths per population size^9^. Such a severe pandemic development and outputs were the main motive for selection of Czechia for a deeper analysis.

CVDs are among the most common causes of death in developed countries and the world. In Czechia (see Table 1 below for more details), before the start of the COVID-19 pandemic, CVDs account for more than 40% of all deaths (both sexes combined). In a longer period, CVDs mortality stably decreased and contributed to improvements in life expectancy. From this point of view, the trend observed in Czechia was comparable to most of the European countries and in accordance with the concept of health transition or cardiovascular revolution^10^.

**Table 1:** Basic data related to the studied period—life expectancy at birth (in years), numbers of death (total, CVDs, COVID-19), numbers of death according to place of death, average monthly provided health care records, Czechia, 2018–2022. Source:^14–16^, author’s calculation.

	2018	2019	2020	2021	2022
Life expectancy at birth (both sexes combined)	79.00	79.24	78.27	77.25	79.01
Total number of deaths	112,920	112,362	129,289	139,891	120,219
Total number of deaths from cardiovascular diseases, % of total	48,792 43.2%	47,393 42.2%	51,299 39.7%	47,873 34.2%	46,248 38.5%
Of which					
at home, % of CVDs deaths	12,818 26.3%	12,501 26.4%	14,230 27.7%	14,539 30.4%	12,912 27.9%
in medical facilities, % of CVDs deaths	27,940 57.3%	26,765 56.5%	27,064 52.8%	24,899 52.0%	24,827 53.7%
in facilities of social care, % of CVDs deaths	6157 12.6%	6420 13.5%	8240 16.1%	6786 14.2%	6930 15.0%
Total number of deaths from COVID-19, % of total	0 0%	0 0%	10,539 8.2%	25,455 18.2%	6035 5.0%
Of which					
at home, % of COVID-19 deaths	x	x	348 3.3%	1294 5.1%	172 2.9%
in medical facilities, % of COVID-19 deaths	x	x	9461 89.8%	23,495 92.3%	5719 94.8%
in facilities of social care, % of COVID-19 deaths	x	x	672 6.4%	505 2.0%	127 2.1%
Average monthly cardiovascular health care provision					
hospitalizations	20,855	20,858	18,178	18,051	18,117
ambulant care	235,938	248,073	242,633	275,988	273,868

The main public health objective is to reduce the impact of the pandemic on society as much as possible. However, before effective medical prevention and treatment of COVID-19 were developed, the introduction of government’s non-medical measures brought many important changes to the community, human behavior, as well as health care providers such as curfews, restrictions on the free movement of people, changes in the distribution of beds and hospital staff for isolated infected patients, bans on visits to hospitals and nursing homes. In Czechia during the first months of the pandemic, the preventive measures were rapid and relatively strict in comparison to other European countries (lockdown already in the middle of March 2020 before the first victim of COVID-19 was even registered in Czechia). This was even supported by the responsible behavior of the public in this initial stage (voluntary sewing of masks when they were in short supply etc.). As a result, the spring 2020 pandemic wave was very mild according to the numbers of confirmed infections as well as victims. During autumn 2020, however, important changes came—decreasing respect for restrictions from the public (among other things it started to be considered unnecessary because of the low number of victims during spring 2020) and late implementation of preventive measures and restrictions by the government, including limited access to COVID-19 testing (above all for specific groups of population—disadvantaged by age, health status, or place of living). The vaccination started rather slowly and it was accompanied by the rejection of part of the public. The otherwise very stable healthcare system in Czechia approached (and in some regions even reached) the collapse during the autumn 2020–spring 2021 period. Due to the often-late measures taken, the consequences were more fundamental and the subsequently taken measures were stricter and more longer-term^9^.

The vaccination process started in January 2021. The groups with priority access to vaccination were chronically ill and above all the oldest seniors (aged 80 and older). The vaccine rollout was extended gradually to younger age groups and to everyone except for children until 12 years old in the summer of 2021. As a result, the spring wave seen in 2021 was more severe among younger age groups on average while the observed mortality excess gradually declined for the oldest age group^11,12^. In contrast to the Western and Northern parts of Europe, COVID-19 vaccination coverage remains on average lower in Czechia^13^, especially in the middle and younger ages. However, this approach and the on average more reserved attitude (protests, lack of respect) of the society towards the measures taken, COVID-19 vaccination, and the overall trust in the government and the system are comparable to other countries in the Central and Eastern part of Europe, i.e., countries with communist past^9^.

Table 1 presents the basic relevant descriptive data for the studied period. Before the pandemic, the life expectancy at birth increased slowly, already the first pandemic year brought a significant decrease in its value, and in the second year, the worsening was repeated. In total, during the pandemic, Czechia went through a period of the deepest annual decrease in life expectancy from WWII. In 2022, values of life expectancy at birth returned almost to their pre-pandemic levels. Similarly, there is an abrupt increase in the number of deaths since 2020.

In the pre-pandemic years, the total annual number of deaths in Czechia (regardless of the cause of death) was around 112 thousand, during the pandemic years it increased to 130 thousand deaths in 2020 and almost 140 thousand deaths in 2021. In the last pandemic year, that is, 2022, it was again around 120 thousand deaths. The higher number of deaths in 2022 in comparison to the pre-pandemic period, could be determined among other by population aging.

The proportion of CVDs deaths decreased from 43% before the pandemic to less than 35% in 2021. In 2022 its proportion increased again, however, still it remains lower than in the pre-pandemic period.

COVID-19, as an underlying cause of death, led to more than ten thousand deaths in 2020, which account for approximately 8% of the total. The following year, 2021, was much worse, and COVID-19 caused 25.5 thousand deaths (more than 18%). In the last analysed year, the number of COVID-19 victims decreased significantly, to around six thousand (5%).

## Data and methodology

The demographic-epidemiological cross-sectional study was based on the overall mortality level and its changes in Czechia during the years 2018–2022. It is a secondary data analysis. The analysis focuses on a comparison of three time periods—pre-pandemic development (2018–2019), pandemic years (2020–2021), and the year when the effect of the pandemic started to decrease (2022). See the Supplementary file for the total numbers of death during the studied years.

The numbers of deceased according to age, causes, month, and place of death for the years 2017–2022 (as well as the age structure of the population in the same years) were taken from the database of the Czech Statistical Office^14,15^. The first year (2017) was used only as a basis, the first annual change of the mortality level is calculated for 2017/18. Numbers of provided cardiovascular health care were taken from databases of the Institute of Health Information and Statistics^16^.

In relation to causes of death, all cases are classified according to the underlying cause of death using the ICD-10 classification defined by the World Health Organization (WHO). Nevertheless, it is assumed that some misclassifications could happen during the first months of the pandemic (2020) when no clear rules for coding or definitions were set as discussed in Limitations.

The first step of the analysis is based on a deeper description of overall changes in life expectancy at birth. Life tables were calculated using traditional demographic methods, and their main output, the value of the life expectancy at birth, was studied on the basis of its annual changes as the results are comparable to other research focused on development in other countries. For the needs of the following analysis, the life tables were not smoothed in any way. The annual changes in life expectancy were decomposed into particular contributions of causes of death. We used the method proposed by Pollard^17,18^, explained in detail in many traditional demographic sources (e.g.,^19^). The results of the decomposition are important for a better understanding of the general observed trends and outcomes of the pandemics. Although there are more potential methods and approaches, it was demonstrated that the compared decomposition methods provide the same or comparable results using the same data^20^. Except for the traditional distinguishing of causes of death in the decomposition, we also incorporated information about places of death, because this structural change of mortality is also considered essential and understudied. Because of the focus and scope of this study, the results are presented as contributions aggregated across the age groups. The detailed results according to age are available in the online repository (see Data Availability section).

The second step of the analysis follows the mortality and admission development in relation to CVDs in more detail and so enriches and helps to understand the results of the first analytical step. The data were aggregated into monthly numbers covering the whole study period (January 2018–December 2022). Using the monthly data, it is possible to follow the changes during the period, including development within the studied years, corresponding with epidemiological development, preventive and policy measures etc. We studied not only the changes in mortality but also the relevant health care provision and usage. A cardiovascular hospitalization was defined as admission to a hospital where there was an “in-hospital diagnosis code of diseases of the circulatory system (ICD-10 code category “I”) with an overnight stay”^21^. Moreover, we also followed the development of the amount of cardiovascular ambulant care (as a number of appointments or other services covered by health insurance made by cardiovascular ambulant specialists). Those time series representing the provided and used cardiovascular health care were analysed in the context of the time series of cardiovascular deaths according to a place of death.

Because of the typical seasonality in CVDs mortality, the second step of the analysis is methodologically based on seasonal decomposition of the monthly time series where the observed values can be adjusted for the seasonality pattern and the development can be studied independently. Using this approach, it is possible to separate and estimate three components of the time series: (1) the overall trend (representing the general direction of the series over a long period of time), (2) seasonal factors (repeated pattern of traditional variation within a calendar year), and (3) irregular component (irregular variation above trend and seasonality representing abrupt or exceptional changes in the time series). We used the multiplicative approach, where the time series is considered as a result of the multiplication of particular components, and X-13 methodology of estimation performed in the statistical software SAS, version 6.4 (see e.g.^22,23^, the Supplementary file, or the online repository for more details). Monthly observations were adjusted for the length of a particular month.

Because the trend component represents the overall direction of the time series, the seasonal component stands for regular and annually repeated monthly patterns of development, it is the irregular component that represents any abrupt changes or unexpected development in the time series that cannot be tied to average seasonality or trend. Values of this irregular component are independent of the trend, seasonal variation, or different lengths of months, and they draw attention to periods of extraordinary development. Values of the seasonal and irregular components that are above one stand for periods (months) with numbers of observations that are above the average trend (seasonal component) or expected values for a specific month (irregular component).

In the time series analysis, we studied in total five series—two representing the development of health care provision (amount of CVDs ambulant care and number of CVDs hospitalizations) and three series for the development of CVDs mortality (CVDs deaths at home, in facilities of social care, and in medical facilities).

## Results

In the analysis, we distinguished four potential types of places of death: deaths at home, in medical facilities (hospitals or other medical facilities), in facilities of social care (social care houses), or at other places. Most CVDs deaths occurred in medical facilities (more than 50% in all the studied years). Before the pandemic, around 26% of CVDs deaths occurred at home annually. During the pandemic, this proportion increased to more than 30% in 2021. COVID-19 deaths occurred mostly (around 90%) in medical facilities (Table 1).

### Decomposition of life expectancy: contribution of CVDs mortality

For the studied years, Table 2 (pre-pandemic development) and Table 3 (development during the pandemic) show the annual changes in life expectancy at birth and contributions of deaths from CVDs and COVID-19 according to places of death.

**Table 2:** Annual changes in life expectancy at birth, contributions of deaths on cardiovascular diseases and COVID-19, and places of deaths, both sexes combined, Czechia, pre-pandemic development, 2017/2018–2018/2019. Source:^14–16^, author’s calculation.

Both sexes		Period before the pandemic							
		2017/18				2018/19			
Total annual change in life expectancy at birth (in years)		0.05				0.25			
Contribution of cardiovascular diseases (in years)		0.14				0.17			
Contribution of cardiovascular diseases according to places of deaths		At home	Medical facilities (hospital)	Fac. of social care	Other places	At home	Medical facilities (hospital)	Fac. of social care	Other places
	Absolute contribution (in years)	0.03	0.13	− 0.01	− 0.01	0.04	0.12	− 0.01	0.02
	Relative contribution (in %) from cardiovascular diseases	19.78	94.38	− 6.16	− 8.01	22.65	71.42	− 4.87	10.80
Contribution of COVID-19 (in years)		0.00				0.00			
Contribution of COVID-19 according to places of death		At home	Medical facilities (hospital)	Fac. of social care	Other places	At home	Medical facilities (hospital)	Fac. of social care	Other places
	Absolute contribution (in years)	0.00	0.00	0.00	0.00	0.00	0.00	0.00	0.00
	Relative contribution (in %) from COVID-19	x	x	x	x	x	x	x	x
Contribution of other causes (regardless of place of death, in years)		− 0.09				0.07			

Note: graphical visualization of the results is stored in an online repository, see the Data availability section.

**Table 3:** Annual changes in life expectancy at birth, contributions of deaths on cardiovascular diseases and COVID-19, and places of deaths, both sexes combined, Czechia, development during the pandemic, 2019/2020–2021/2022. Source:^14–16^, author’s calculation.

Both sexes		Beginning of the pandemic				The peak of the pandemic			
		2019/20				2020/21			
Total annual change in life expectancy at birth (in years)		− 0.97				− 1.03			
Contribution of cardiovascular diseases (in years)		− 0.18				0.15			
Contribution of cardiovascular diseases according to places of deaths		At home	Medical facilities (hospital)	Fac. of social care	Other places	At home	Medical facilities (hospital)	Fac. of social care	Other places
	Absolute contribution (in years)	− 0.10	0.01	− 0.08	− 0.01	− 0.01	0.09	0.06	0.01
	Relative contribution (in %) from cardiovascular diseases	56.88	− 3.44	43.02	3.54	− 8.71	63.73	39.08	5.90
Contribution of COVID-19 (in years)		− 0.74				− 1.19			
Contribution of COVID-19 according to places of death		At home	Medical facilities (hospital)	Fac. of social care	Oher places	At home	Medical facilities (hospital)	Fac. of social care	Oher places
	Absolute contribution (in years)	− 0.03	− 0.67	− 0.03	− 0.01	− 0.09	− 1.09	0.01	− 0.01
	Relative contribution (in %) from COVID-19	4.39	90.24	4.67	0.70	7.78	91.66	− 0.44	1.00
Contribution of other causes (regardless of place of death, in years)		− 0.04				0.01			

		Ending of the pandemic							
Both sexes		2021/22							
Total annual change in life expectancy at birth (in years)		1.76							
Contribution of cardiovascular diseases (in years)		0.20							
Contribution of cardiovascular diseases according to places of deaths		At home	Medical facilities (hospital)	Fac. of social care	Other places				
	Absolute contribution (in years)	0.13	0.07	0.00	0.01				
	Relative contribution (in %) from cardiovascular diseases	61.60	34.89	0.83	2.68				
Contribution of COVID-19 (in years)		1.54							
Contribution of COVID-19 according to places of death		At home	Medical facilities (hospital)	Fac. of social care	Other places				
	Absolute contribution (in years)	0.11	1.39	0.02	0.02				
	Relative contribution (in %) from COVID-19	7.4	90.3	1.3	1.0				
Contribution of other causes (regardless of place of death, in years)		0.02							

Note: graphical visualization of the results is stored in an online repository, see the Data availability section.

Life expectancy at birth is a summary measure of the current health status of the population under study. If the level of mortality increases, life expectancy decreases, and vice versa. According to contributions of particular causes of death, if mortality from the selected cause increases, the contribution of this cause to a change in life expectancy is negative, i.e., worsening of mortality from any cause of death negatively contributes to a life expectancy change.

Between the years 2019 and 2020, the life expectancy at birth decreased by almost a year. The second pandemic year brought a further decrease in life expectancy, by more than a year (− 1.03 years). The year 2022 was the first year since the pandemic during which life expectancy increased again (+ 1.76 years).

Before the pandemic, a decrease in CVDs mortality contributed significantly to the growth in life expectancy (Tables 2 and 3). Between the years 2017 and 2018, the contribution of CVDs was even higher than the overall increase in life expectancy (+ 0.14 and + 0.17 years; the positive contribution of CVDs was moderated by a mortality increase from other causes of death).

A significant change was observed in the first pandemic year—the contribution of CVDs was negative (− 0.18 years; mortality from CVDs increased) and supported the negative development of life expectancy in 2020. Thus, the higher level of CVDs mortality in 2020 contributed to the overall reduction in life expectancy at birth (Table 3).

In the second year of the pandemic, there was a reversal. Despite the COVID-19 pandemic peaking in 2021, the positive development in CVDs mortality was observable. Improvement in CVDs mortality helped to moderate the life expectancy decrease caused mostly by COVID-19. In 2022, also CVDs contributed significantly (+ 0.15 years) to the overall rapid increase in life expectancy (Table 3).

The next part of Tables 2 and 3 describes the contribution of COVID-19 to life expectancy changes. In the first year of the pandemic, COVID-19 mortality led to a reduction in life expectancy at birth by 0.74 years. The second pandemic year was even worse, and COVID-19 itself led to a decrease in life expectancy by 1.19 years. In the final year of the pandemic, mortality from COVID-19 decreased again.

Tables 2 and 3 show also the contribution of CVDs and COVID-19 mortality according to places at death. Most of the negative contribution of COVID-19 was due to deaths in medical facilities because most of the COVID-19 deaths occurred in hospitals (90–92%). Vaccination against COVID-19 was initiated during the first months of 2021, preferably from the oldest age groups, or in facilities of social care. This is reflected in the positive contributions of COVID-19 mortality at facilities of social care to life expectancy change between 2020 and 2021.

The situation was different according to CVDs. Before the pandemic, contributions of CVDs mortality were around zero at all places except for medical facilities where decreasing CVDs mortality helped to increase the overall life expectancy. Whereas the CVDs mortality rates in hospitals improved until 2019, CVDs mortality rates out-of-medical facilities were almost stable (Table 2).

In the first pandemic year (2020), the negative contribution of CVDs mortality to the overall change in life expectancy was mainly due to higher CVDs mortality at home and in social care houses. At these two places, the level of CVDs mortality worsened, i.e., the number of CVDs deaths at home or social care houses increased the most. In 2021, CVDs mortality in social care houses and in medical facilities improved and helped to decrease CVDs mortality. The contribution of CVDs mortality at home was already close to zero, but still negative (supporting the decrease in life expectancy). In the final pandemic year, 2022, contributions of CVDs mortality regardless of the place of death were positive again (Table 3).

### Seasonal decomposition: identification of months with specific development according to a place of CVDs mortality

**Figure 1:**
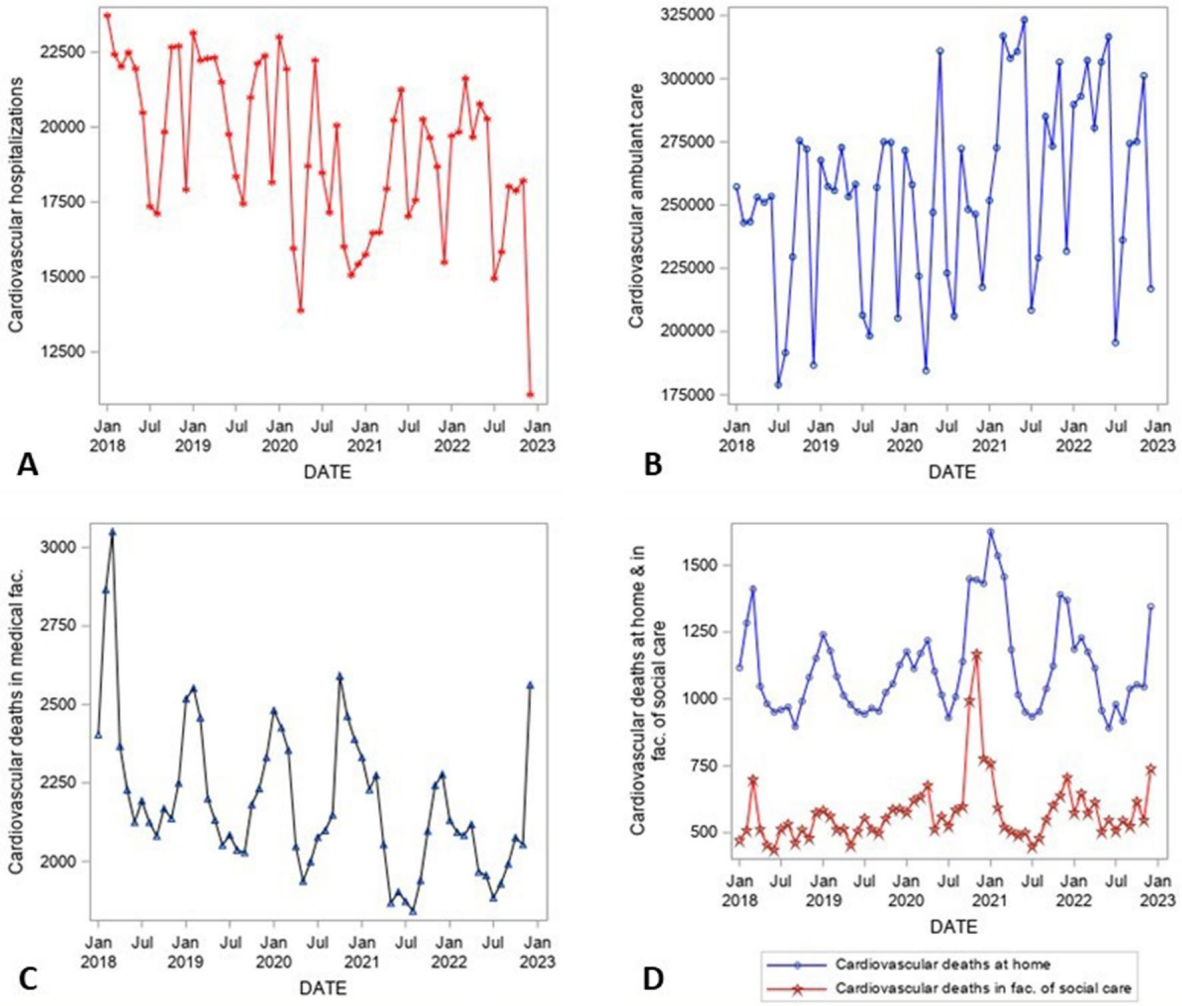
Time series of monthly data adjusted for the length of particular months, January 2018–December 2022, CVDs hospitalizations (**A**), CVDs ambulant care (**B**), deaths in medical facilities (**C**), deaths at home and facilities of social care (**D**), Czechia. Source:^14–16^, author’s calculation, output of the SAS software, version 6.4.

**Figure 2:**
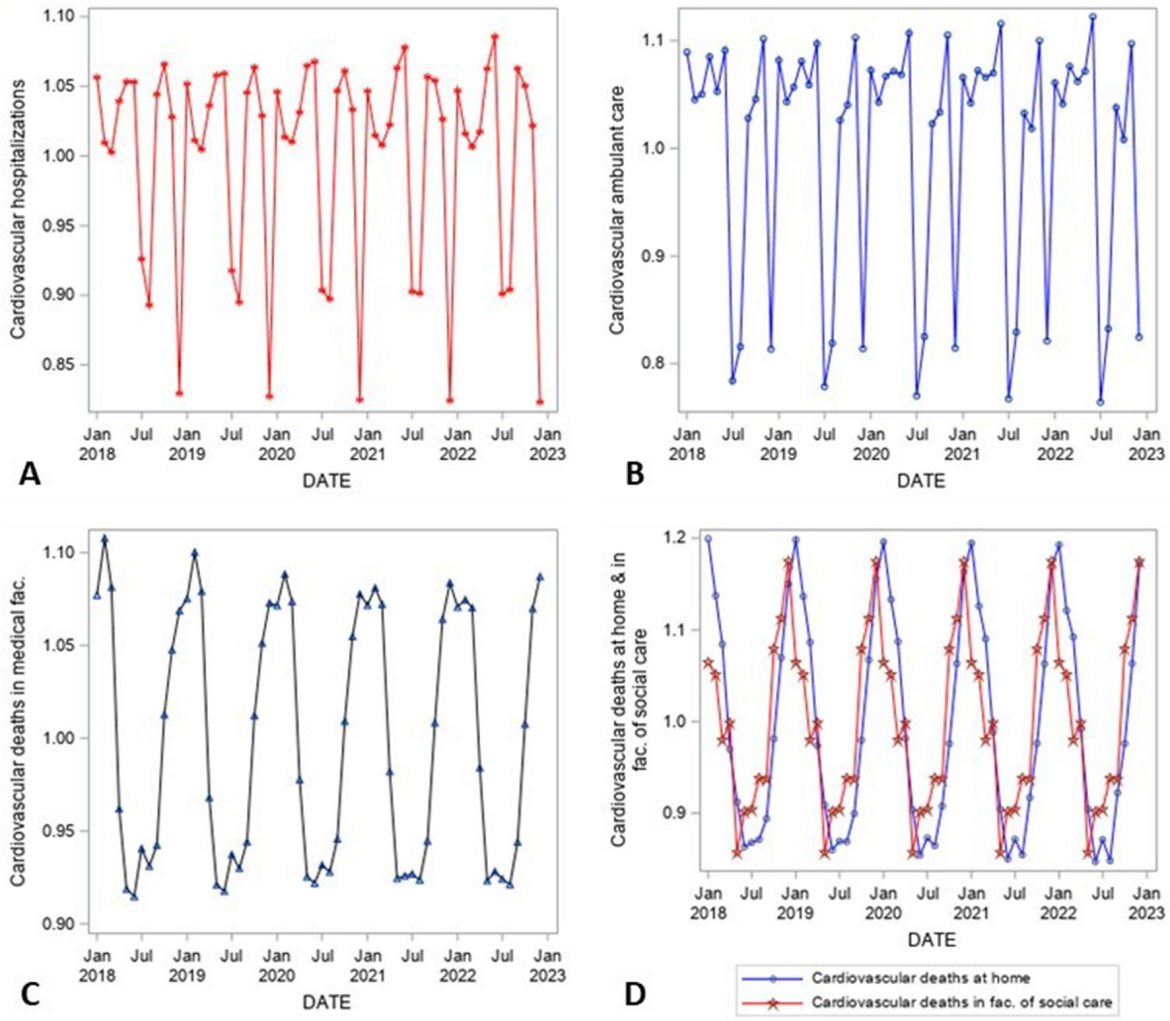
Seasonal component of time series decomposition (1 = average month corresponding to the overall trend of the time series), January 2018–December 2022, CVDs hospitalizations (**A**), CVDs ambulant care (**B**), deaths in medical facilities (**C**), deaths at home and facilities of social care (**D**), Czechia. Source:^14–16^, author’s calculation, output of the SAS software, version 6.4.

**Figure 3:**
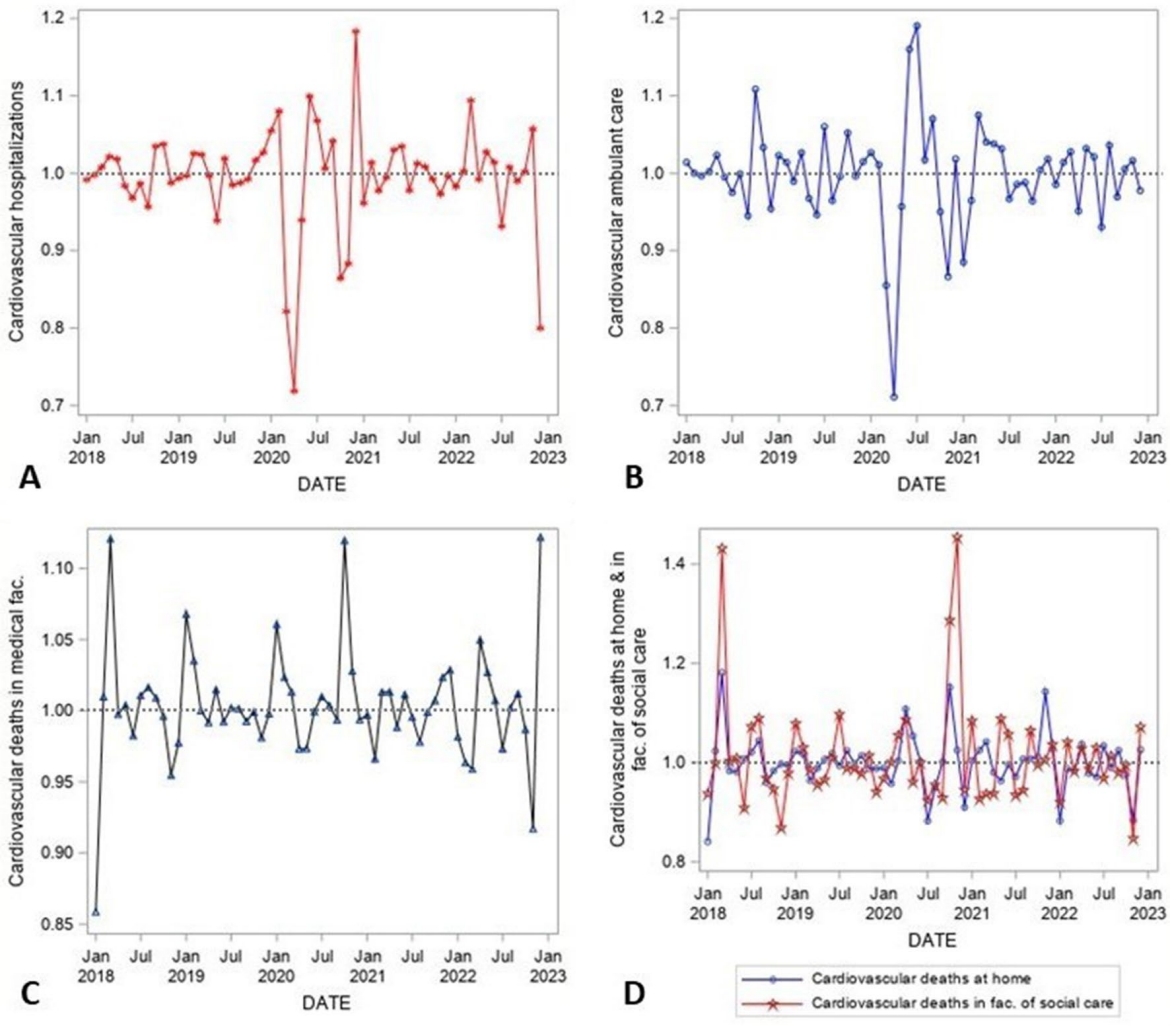
Irregular component of time series decomposition (1 = average month corresponding to the overall trend and seasonal factor of the particular month of the time series), January 2018–December 2022, CVDs hospitalizations (**A**), CVDs ambulant care (**B**), deaths in medical facilities (**C**), deaths at home and facilities of social care (**D**), Czechia. Note: the dotted horizontal line at value one represents the reference level corresponding to an expected values of the time series for a particular month reflecting the trend and seasonal pattern. Source:^14–16^, author’s calculation, output of the SAS software, version 6.4.

Figure 1 shows the overall development of CVDs health care provision and mortality in Czechia in time, i.e., development of the studied time series—CVDs hospitalizations (panel A), CVDs ambulant care (B), CVDs deaths in medical facilities (C), at home, and in facilities of social care (D). The monthly data are adjusted for the length of particular months in the period January 2018–December 2022. Clearly, in the time series, a strong seasonal pattern can be seen (see Fig. 2—Seasonal component of time series decomposition). There is also a long-term decreasing trend in some series—above all CVDs hospitalizations (Fig. 1A) or CVDs deaths in medical facilities (Fig. 1C). This trend started already before the pandemic. On the other hand, there was an increase in CVDs ambulant care before the pandemic, which was interrupted in 2020 (Fig. 1B). The number of CVDs deaths at home or in facilities of social care had almost a stable trend during the studied period, however, with high variability (Fig. 1D). The visible peak at the end of 2020 could be considered as an indirect effect of the pandemic and will be discussed later in more detail. The significant exceptional increases or decreases in the development are depicted in Fig. 3.

The seasonal patterns (Fig. 2) of the analysed series are stable in time (in contrast to more traditional approaches to time series seasonal decomposition, the used X-13 methodology potentially allows for moving seasonal components, slightly developing in time). Values around one represent an average month within a year. There is a traditional decrease in the number of CVDs hospitalizations as well as CVDs ambulant care during the summer and in December each year (by some 20%, represented by values around 0.8 in Fig. 2). On the other hand, the peak of CVDs mortality repeats annually in the first quartile of the year (regardless of the place of death).

Figure 3 shows the irregular component of the time series decomposition. It reveals the unexpected or exceptional changes in the studied time series. Values around one correspond to expected values of the time series for a particular month reflecting the trend and seasonal pattern. For CVDs health care, there is an abrupt drop in March and April 2020, in both time series the values of provided health care decreased in those months to almost 70% of expected values (values around 0.7 in Fig. 3). This decrease is partially replaced by an exceptional increase in CVDs ambulant care during the summer of 2020, however, this increase was only about 20%. Another decrease in the number of CVDs hospitalizations as well as ambulant care was observable at the end of 2020. The observable decrease in CVDs hospitalizations at the very end of 2022 may correspond to the strong flu epidemics at that time.

Several peaks of CVDs mortality were seen during the studied years. The first peak occurred at the beginning of 2018. This likely corresponds to weather conditions and flu epidemics typical for this time of the year, however stronger in the studied year. Except for these smaller or bigger peaks repeating similarly (however, not strictly regularly so as to be included in the seasonality pattern) every year during the first months (reinforcing the long-term seasonal effect), there are no strong deviations from random fluctuations around one (corresponding to average months). The change occurred with the start of the pandemic—in April 2020, there was an increase in the number of CVDs deaths at home by 10% in comparison to an average April in the studied years and an increase of almost 9% in deaths in facilities of social care. This corresponds to the observed decrease in CVDs health care or hospitalization in March or April of that year. For the sake of completeness, this time, from March 12th to May 17th, 2020, a State of Emergency was declared in Czechia^24^.

An even more significant increase in CVDs deaths occurred, however, at the end of 2020—above all in October 2020 (increase by 12% in medical fac., 15% at home, and 29% in facilities of social care) and November 2020 (increase by 45% in facilities of social care, the increase in deaths at home or in medical facilities was around 2–3%). At this time, the next State of Emergency was declared in Czechia in the period from October 5^th^, 2020, to February 14^th^, 2021^24^.

The last exceptional increase in deaths at home occurred in November 2021 by 14% and at medical facilities (hospitals) in December 2022 by 12% which likely corresponds to an already mentioned flu epidemic.

## Discussion

This research analysed the effects of CVDs and COVID-19 mortality on annual life expectancy changes in Czechia. The analysis introduces the possibilities of two methodological approaches and their importance for a deeper understanding of the observed development and its evaluation. The first step is a traditional demographic method of decomposition of the changes in life expectancy at birth. In the study, less traditionally also the place of death is considered. The second step uses the monthly time series, which enables a detailed study of the development during the studied years and helps to identify the particular months of exceptional changes. The used method of seasonal decomposition adjusts the observed trends for an important seasonal pattern. The time series analysis helps to enrich and explain better the results from the decomposition, clearly the mortality changes are concentrated only in several months during the studied years.

Results of the presented decomposition, on changes in mortality patterns by CDVs and COVID-19 and according to a place of death during the pandemic in Czechia, confirm and conform to other published studies. However, most relevant publications do not focus on the situation in the Central and Eastern European region, where the pandemic had an extremely serious impact and the non-medical preventive measures were exceptionally strict and common.

In relation to the health status of the population as well as continuing population aging in the developed world^25^, many authors suppose that during the pandemic, some sub-population in need of acute health care might have stayed at home and did not attend the emergency or hospital in case of need^5,6,21,26^.

In our study, the abrupt and deep decrease of CVDs health care provision was confirmed, above all during the first phases of the pandemic. This is in line with the development observed in other states as well^3^. Already in Spring 2020, as a result of a necessary reorganization of the healthcare system, in many countries the non-vital procedures were often postponed or cancelled, and personal meetings were replaced by telemedicine, where possible^21^.

It can be supposed that there could be direct consequences of this limited health care for patients suffering from acute CVDs^2,3,27^. In the Duke University Health System, a 33.1% decrease in the number of cardiovascular visits during the first 15 weeks of the pandemic was recorded^27^. In the U.S., across a tertiary health care system, the estimated decrease in the number of hospitalizations in March 2020 in comparison to March 2019 was 43.4%^28^. The decreasing trend of hospitalizations was observed also in Germany^29–31^. In Czechia, the observed monthly decrease of CVDs hospitalizations as well as ambulant care was almost 30% in comparison to average trend in March and April 2020.

Most of the authors, however, estimated the overall risk of cardiovascular death during hospitalization as completely or almost unchanged. In Czechia the CVDs mortality increased, the number of CVDs deaths was by some 10% higher already in April 2020. However, the increased number of CVDs deaths was registered mostly at home or facilities of social care, the CVDs mortality in medical facilities remained more stable. Similar structural changes according to places of death were observed also in other countries and other studies^5–7,21,32,33,36^. In all the studied populations, the increase of CVDs mortality is related to deaths at home. For Czechia, the results above confirmed the trend also in facilities of social care what is not always studied in another research.

The supposed mechanism of the relationship between COVID-19 and cardiovascular care, prevalence, and mortality was illustrated by Raisi-Estabragh and Mamas^35^. According to them, during the pandemic, the diagnosis as well as the treatment of CVDs were often delayed. They also warned that the prevention possibilities were lower, and even acute care was limited. This indirect impact of the pandemic was even called a crisis in healthcare systems^34^. Moreover, the social healthcare inequalities may have also increased^35^.

Although it can be supposed that a mortality worsening was a consequence of delayed seeking and provision of medical care^5^, some authors identified the sources of the development of cardiovascular care provision and cardiovascular mortality on both sides^2^—fear of the patients (leading to the decrease in demand for acute health care) and changes in the management and organization of health care (leading to a decrease of supply of medical care, postponement of care, etc.). However, only from the total numbers of provided CVDs health care, it is difficult to distinguish the demand and supply sides of the heath care market.

Some authors^6^ also questioned the possibility of undiagnosed COVID-19 victims being classified as cardiovascular deaths. This may have played a role above all in the first stages of the pandemic where access to COVID-19 testing was limited, and definitions were unclear. Based on the presented results, we can conclude that the observed increase of CVDs mortality corresponds not only to limitations in the provided health care, but also to peeks of the pandemic, it cannot be strictly tied only to health care limitation. Moreover, the increase of CVDs mortality above all at home or in social care facilities, i.e., out of medical care and testing possibilities, corresponds to the assumption of potential misclassification of the causes of death.

Although this paper focused specifically on CVDs during the pandemic years, there might have been a significant impact also on cancer incidence and mortality. Delayed diagnosis may have resulted in the development of illnesses because of postponed health care or preventive screening^26^. The impact of such delay, however, can be studied and understood only in the future.

## Limitations of the study

In the study, we worked with cases of death that are classified according to the underlying cause of death. However, there might be inconsistencies in the coding practice as the pandemic evolved over time as well as in the administrative recommendations for the classification of the deaths and causes of deaths. We have to assume that above all during the initial phases of the pandemics there could be an increase of registered CDVs deaths not only because of higher CVDs mortality but also as an impact of unrecognised COVID-19 cases. Logically, this may be important aspect related above all to deaths at home or in social care facilities.

In the analysis, we used the seasonal decomposition of the time series. Above all, the irregular component showing exceptional increases or decreases in the time series might be difficult for understanding, as it does not fully visibly correspond to the observed time series of the input data. Still, we prefer this approach because the irregular component represents the exceptional changes above the trend as well as the traditional seasonal variation. In other words, the irregular component represents the variation in the time series adjusted for the trend development and typical seasonal pattern.

## Conclusion and implications

As could be expected, during the two pandemic years, the main cause of death contributing to the decline in life expectancy at birth was COVID-19. However, in the first year of the pandemic, in 2020, the decline in life expectancy was only partially explained by the mortality rate from COVID-19, also the CVDs mortality contributed significantly to the overall mortality worsening. As the increase in CVDs deaths during the autumn of 2020 was much higher than the decrease in provided CVDs health care in the same months, we can formulate a hypothesis that part of the CVDs mortality in that time may correspond to the limited CVDs care during the spring, but also to inaccurate coding of causes of death.

The life expectancy decomposition approach and time series analysis, focusing on a more in-depth study of CVDs during a pandemic, presented above, provide public policy authorities with an important evidence base on where the attention or prevention needs to be targeted. Only such a deeper knowledge of the important consequences and influencing factors could more efficiently help to avoid a systemic risk of insufficient or late health care with fatal outputs or public health damage during any potential future health crises or other challenges leading to a change in the organization of the health care system or in people's behavior^6^. It is important to be aware of the sensitivity of CVDs in prevention and prompt and acute health care.

In Czechia, the most significant declines in CVDs healthcare provision and increases in CVDs mortality occurred in the time of State of emergency. For cases of any potential future crises affecting people’s behavior or healthcare usage, it is crucial to ensure the availability of acute healthcare as well as support for the population's awareness of the fundamental role and irreplaceability of acute healthcare in the protection of public health.

### Supplementary Information

Supplementary Information.

## Data Availability

Data used in this study for the analyses are partly publicly available. The unpublished mortality data were provided for scientific purposes by the Czech Statistical Office in a de-identified individual-level form (the source is cited in the text). Monthly time series of provided cardiovascular ambulant care and cardiovascular hospitalizations were provided upon request by the Institute of Health Information and Statistics (https://www.uzis.cz/index-en.php?pg=contact). All the following calculations were prepared in MS Excel using the methods described in the text of the paper and in the SAS Software, version 9.4. The detailed results of the decomposition as well as the code for the SAS software is provided in the online repository: https://github.com/klarahulikova/Impacts_of_COVID_19_DECOMP. Ethical approval was not required for this secondary analysis.
